# Alcohol and NMDA receptor: current research and future direction

**DOI:** 10.3389/fnmol.2013.00014

**Published:** 2013-05-28

**Authors:** Raman Chandrasekar

**Affiliations:** Department of Biochemistry and Biotechnology Core Facility, Kansas State UniversityManhattan, KS, USA

**Keywords:** alcohol, glutamate, NMDA receptor, fetal cortical neurons, splice variant, epigenetic, transcription, RNA-binding protein

## Abstract

The brain is one of the major targets of alcohol actions. Most of the excitatory synaptic transmission in the central nervous system is mediated by N-methyl-D-aspartate (NMDA) receptors. However, one of the most devastating effects of alcohol leads to brain shrinkage, loss of nerve cells at specific regions through a mechanism involving excitotoxicity, oxidative stress. Earlier studies have indicated that chronic exposure to ethanol both *in vivo* and *in vitro*, increases NR1 and NR2B gene expression and their polypeptide levels. The effect of alcohol and molecular changes on the regulatory process, which modulates NMDAR functions including factors altering transcription, translation, post-translational modifications, and protein expression, as well as those influencing their interactions with different regulatory proteins (downstream effectors) are incessantly increasing at the cellular level. Further, I discuss the various genetically altered mice approaches that have been used to study NMDA receptor subunits and their functional implication. In a recent countable review, epigenetic dimension (i.e., histone modification-induced chromatin remodeling and DNA methylation, in the process of alcohol related neuroadaptation) is one of the key molecular mechanisms in alcohol mediated NMDAR alteration. Here, I provide a recount on what has already been achieved, current trends and how the future research/studies of the NMDA receptor might lead to even greater engagement with many possible new insights into the neurobiology and treatment of alcoholism.

## Introduction

Unhealthy alcohol consumption remains a main problem for the public health and is responsible for a high rate of morbidity, affecting various organ systems and mortality. In mammals L-glutamate is known to have a role in regulating brain maturation processes during the pre- and postnatal period of developmental at the morphological (neuron proliferation and differentiation, synaptogenesis), functional (post-tetanic potentiation and depression), and behavioral (learning and memory) levels. The N-methyl-D-aspartate (NMDA) subtype of glutamate receptor is involved in fast excitatory synaptic transmission and neuronal plasticity in the central nervous system (Loeches and Guerri, 2011). Chronic ethanol exposure has complex and long-lasting effects on the function and/or expression of a myriad of neurotransmitter receptors and their modulators (Lovinger, 1997). A group of proteins affected by chronic ethanol exposure are ligand-gated ion channels such as the glutamatergic ionotropic receptors. The evolution of ionotropic glutamate receptors' subunits (NR1, NR2, NR3) proteins are remarkably well-conserved between species from lower organism to mammals, consistent with the importance of NMDARs in nervous system function (Ewald and Cline, 2009; Tikhonov and Magazanik, 2009; Koo and Hampson, 2010; Teng et al., 2010; Witt, 2010; Flores-soto et al., 2012). The physiological and pharmacological properties of glutamate activate three classes of glutamate-gated ion channels (AMPA, Kainate and NMDA receptors) which transduce the postsynaptic signal. In particular, NMDA receptors have higher affinity for glutamate, slower activation and desensitation kinetics, higher permeability for calcium and of particular importance, susceptibility to potential-dependent blockage by magnesium ions. The functional NMDA receptors are multiple heterodimer complexes composed of NR1 combined with one or more NR2 (NR2A, NR2B, NR2C, NR2D) subunits and less commonly NR3 (A, B) subunits (Kaniakova et al., 2012). Homology is about and is significantly greater in the conserved functional domains, for example at the C-terminal (Tikhonov and Magazanik, 2009). The genes are expressed in various tissues and brain regions and are likely to be under complex expression control. In addition to up regulating NMDA receptor subunit expression, chronic ethanol also increases NMDA receptor functionality (i.e., conductance cation influx) and synaptic clustering of the receptor. The neuroadaptational changes induced by exposure to alcohol and drugs of abuse may be related to dysregulation of signaling systems, gene transcription, translation, and protein expression at the cellular level. Thus, the search for molecular mechanisms that contribute to the initiation and maintenance of the alcohol addictive process has become a major focus of the neuroscience of alcoholism.

A substantial scientific effort has been directed toward increases in NMDA receptor subunit levels contributing to up-regulation of NMDA receptor subunits gene expression (glutamate transmission) by ethanol exposure in adult brain and fetal cortical neuron cells (Anji and Kumari, 2006, 2011). Although ethanol primarily acts on ion channels, there is also evidence that ethanol alters Group I mGlu receptor functions. Nonetheless, AMPA/KA receptors in the central amygdala have recently been reported to be important for the conditioned rewarding effects of ethanol (Loeches and Guerri, 2011; Moykkynen and Korpi, 2012). Evidence is emerging that there are clinical and experimental studies which have provided direct evidence that the adult and developing brain is vulnerable to the toxic effects of alcohol and that alcohol abuse during pregnancy can cause permanent brain damage in offspring which is associated with life-long behavironal, social, and cognitive disorders (White et al., 2011). However, new emerging approaches for novel epigenetic regulation of gene expression in the context of alcohol are more interesting in the field of neuronal disorder. There is no doubt about the progress that has been made in linking the behavioral effects of alcoholism to the underlying neural mechanism. But there is still a significant gap in our understanding of the exact molecular mechanism allied with the alteration in gene transcription due to the epigenetic mechanism and altered function on transcription factors. Hence the attempt was made to emphasize the effects of alcohol on a cell and molecular level (transcription, translation and post-translation modification) associated with epigenetic regulation.

This review illustrates the power of NMDA receptor subunits past and recent to advances knowledge of the cellular and molecular mechanism participating in the effect of alcohol on the adult and developing brain. I will describe in the first part of this review, the molecular structure and functions of NMDA receptor subunits. The second part will describe the alcohol effect of brain shrinkage and a high-tech-tool for pre-clinical detection. The third part will focus on recent advances of the alternative splicing variant of NMDA receptor subunits. The fourth part will delineate our ignorance to show how far we are from a proper understanding of the ethanol effect of major molecular events like signaling pathway, transcriptional, translation and post-transcriptional modification. Lastly, I will provide a “road map” of the latest development of the epigenetic mechanism of the NMDA receptor.

### Molecular structure of ionotropic NMDA receptor

**Figure 1:**
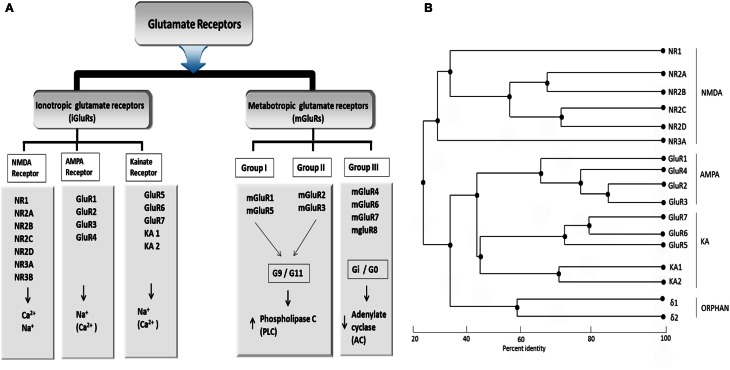
**(A)** Classification of glutamate receptor **(B)** Phylogenetic analysis of all Glu receptor protein membrane. Sequence similarity of ionotropic and metabatropic gluatamate receptor family members. Branch length reflects distances between sequences. The bar indicated the normalized distance score derived from the pairwise sequence similarity score according to Feng and Doolittle, 1987. PLC, Phospholipase C; AC, Adenylaste cylase; downwards arrow, decrease; upwards arrow, increase concentration. Adapted from Kew and Kemp (2005).

Based on pharmacological specificity, members of the iGlu receptor family (Figure 1A) can be classified as either belonging to the NMDA class or the non-NMDA class [α-amino-3-hydroxy-5-methyl-4-isoazolepropionic acid (AMPA receptors) and 2-carboxy-3-carboxymethyl-4-isopropenylpyrrolidine (kainite receptors)]. In humans, seven of the iGlu receptor subtypes belong to the NMDA class (NR1, NR2A–NR2D, NR3A, NR3B) while the remaining belongs to the non-NMDA class. The non-NMDA class could be further divided into the AMPA (GluA 1–4), Kainate (Glu5–Glu7), and delta subtypes (δ1 and δ2), which are sometimes referred to as orphan receptors as it is unclear which ligands they bind and whether they could form functional ion-gated receptors. Native receptors of all these families are likely teteromeric assemblies comprising more than one type of subunit. Although the average of overall amino acid identity of inotropic glutamate receptor subunits across the three families is only about 30%, they share common structural features, which clearly place them into a single large superfamily (Figure 1B). The NMDA receptors require glutamate and glycine (and/or D-serine) for activation, whereas non-NMDA receptors are activated by glutamate alone.

**Figure 2:**
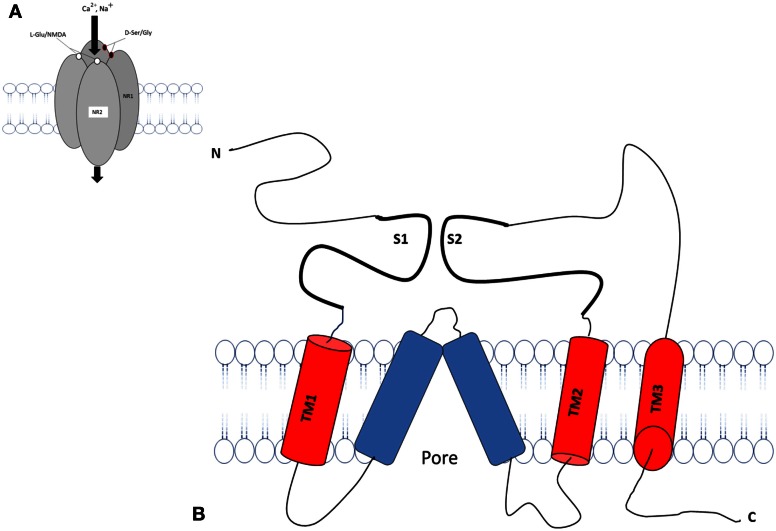
**Schematic diagram of an individual subunit of iGlu receptor. (A)** Proposed membrane topology of an individual iGluR subunit (NR1/NR2). **(B)** The ligand-binding region in the iGluR is formed by two separate extra cellular loops containing the S1 and S2 domains. There are three hydrophobic trans membrane domains, TM1, TM2, and TM3 which fully span the membrane. A re-entrant membrane loop forms the pore that lines an ion channel in iGlu receptors. The amino terminal domain (N) and the ligand binding domain are located in extracellular space. The carboxy-terminal (C) domains situate intracellular and regulatory activity. The model was adapted and modified from Dingledine et al. (1999), Koo and Hampson (2010).

**Figure 3:**
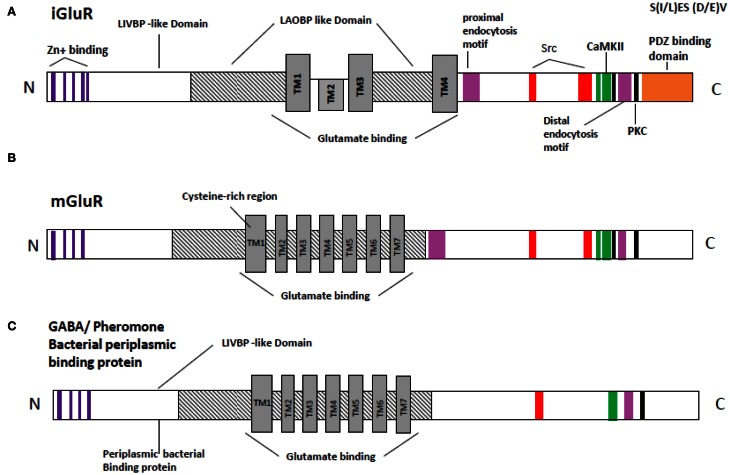
**Schematic representation of the relation sequence conservation with in functional domains of NMDA receptor (GluR) with bacterial periplasmic receptor. (A)** Inotropic glutamate receptor **(B)** metabotropic receptor **(C)** bacterial periplasmic binding protein or/GAGA receptor. Orange, PDZ binding domain [according to Christopherson et al. (2001) and Xia et al. (2000)]; black, PKC; green, CaMKII [according to Strack et al. (2000)]; Red, Src binding domain [according to Schumann et al. (2009) and Nakazawa et al. (2001)]; Lavender, proximal endocytosis motif; Delphinium, Zing binding domain; TM1–TM7, Trans membrane domain 1–7; LAOBP, lysine-arginine-ornithine binding protein; LIVBP, Leucine/isoleucine/ornithine binding protein region are predicated accordingly Ryan et al. (2008).

The transmembrane topology of the Ionotropic glutamate subunits possesses an extra cellular amino terminal domain, which exhibits homology to the mGlu receptor bi-lobed agonist binding domain, followed by a first transmembrane domain (TM1) and then a pore forming membrane-residing domain that does not cross the membrane but forms a re-entrant loop entering from and exiting to the cytoplasm. The second (TM2) and third transmembrane domains (TM3) are linked by a large extracellular loop and the third transmembrane domain is followed by an intracellular carboxy-terminus (Figures 2A,B). Currently crystal structures of the S1–S2 iGlu receptor domains are available for GluN2, and NMDA NR1 receptors in different agonist and antagonist bound forms (Armstrong and Gouaux, 2000). The iGlu receptor ligand binding domain, which is comprised of polypeptides in both the amino terminus (S1 domain: SYTANLAAF 647–655 aa) and the extracellular loop between transmembrane domain 3 and 4 (S2 domain: SYTANLAAF), has confirmed this topology model for the iGlu receptor family (Yao et al., 2008; Koo and Hampson, 2010). The currently accepted mechanism of iGlu receptor channel dynamics is that ligand-induced closure of S1–S2 domains led to conformational changes that triggered iGluR activation (McIlhinney et al., 2003; Oswal et al., 2007). Interestingly, it was reported that the mGlu receptors Venus flytrap domain as well as the iGlu receptor's N-terminal domain shared sequence homology to the leucine/isoleucine/valine-binding protein (LIVBP) of the bacterial periplasmic binding protein (PBPs) (Marinelli et al., 2007). It has been reported that the S1–S2 domain in iGlu receptors forms a pocket that shares mechanistic similarity with PBPs in ligand recognition (Koo and Hampson, 2010). Therefore, it is reasonable to ask whether a common primordial receptor existed and if so, was it more similar to the iGlu receptors or the mGlu receptors, especially within the smaller Venus flytrap domain in the extracellular region common to both receptors. Both iGlu and mGlu receptors share sequence homology with the bacterial PBPs (Figures 3A–C). The modular organization of Glu receptors, especially for the ligand binding domain and the ion channel, has facilitated the study of their structure-function relationship.

### Transmembrane domain evolution

The mosaic or modular proteins like Glu receptors occurred in many eukaryotic proteins, at the time of metazoan radiation. Each module or domain is encoded by a different exon, but the exon-intron boundaries are not coincident with the functional sites of the domain described (ATD, LBD, ion channel). One possibility is that the original intron pattern is hidden by deletion and insertion of introns during the evolutionary history of Glu receptors. The Glu receptors' evolution might have come by the splicing of a primordial channel structure into a prokaryotic periplasmic amino acid binding protein (Figure 3C) M4 sequence analysis for the Glu receptor family shows conservation of the packing interface identified and the residue identity is maintained within the subfamilies. Additionally, conservative substitutions in and around the packing residues can be observed between subfamilies (Figures 1B, 3A–C). M4 coevolution with the core of the ion channel must have shaped this divergence between subfamilies (Koo and Hampson, 2010). In NMDA receptors, the more intracellular segments of M1 have high variability. This might be an indication of the specific coevolution with M4. On the other hand, the intracellular half of the M3 segment is also quite variable. In the case of GluA1–4 receptors, M1 and M3 segments are identical. M4 has two conservative substitutions outside of the packing face. It also will imply that the subunit composition of GluA1–4 receptors is determined mainly by other domains in the subunits because the ion channel can be promiscuous. Complete characterization of each domain is unlikely to result in simplistic discreet roles as alluded to above, but rather to complex, overlapping multi-functional roles. Species variations in receptor pharmacology complicate the study further, making it difficult (at this time) for interspecies comparisons to be made. However, targeting the specific interaction between trans membrane helices of Glu receptor can be a method of disrupting receptor function for application in drug design and structure function studies of the receptor.

### Metabotropic glutamate receptors

Based on their amino acid sequence homology, pharmacological profile and second messenger coupling, metabotropic glutamate receptors fall into three groups. mGluR I (mGluR1–R5) is linked to phospholipase C activation causing an increase in inositol triphosphate concentration and Ca^+^ immbolization. Group II (mGlu2–3) and III (mGluR4–8) receptors inhibit adenylyl cylase activity, resulting in a fall in intracellular cAMP concentration. Physiologically, group I receptors are usually associated with excitatory synaptic responses whereas group II and III are associated with depression of synaptic responses, via inhibition of glutamate release (Gu et al., 2012).

### Non-NMDA ionotropic glutamate receptors

**Figure 4:**
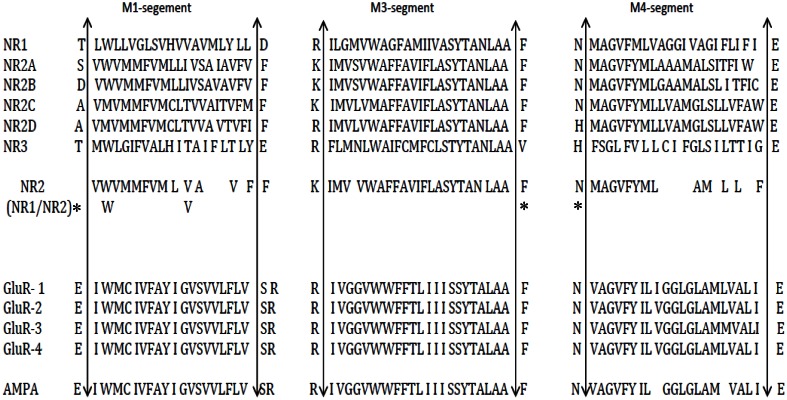
**Comparative sequence analysis of the trans membrane domain (M1,M3,M4) region of NMDA receptor subunits, Glu receptor with AMPA.** Upward arrow indicates the trans membrane region end; ^*^indicates the consensus residue of NR1/NR2 subunits. [Adapted from Higuera (2009)].

AMPA receptors are their homomeric or heteromeric oligomers composed of GluA 1–4 subunits and mediate rapid postsynaptic glutamatergic neurotransmission. Differences in the functional properties of native AMPA receptors are the consequence of different assembles of these subunits. Both homomeric and heteromeric receptors have high affinity for AMPA and low affinity for kainite (reviewed in Bettler and Mulle, 1995). Kainate receptors are composed of five subunits: GluR5–7 and KA1–2. The subunits GluR5–7 have moderate degrees of homology (30–40%) to the AMPA receptor subunits and are components of high affinity kainite receptors. Interestingly GluR7, KA1, and KA2 fail to form channels when each protein is expressed alone, but contribute to heteromeric assemblies of functional kainite receptors when expressed with GluR5 and GluR6. The co-expression of GluR5–7 with KA1 and KA2 in different combinations, leads to the formation of channels with unique properties that resemble neuronal kainite receptors. The Q/R (glutamine/arginine) site of the AMPA and kainite receptor subunits (Kung et al., 2001) is collectively referred as the Q/R/N site (Figure 4). High Ca^2+^ permeability and a voltage dependent blockade of NMDA receptors are critically dependent on the presence of asparagine at this site. Although both NR1 and NR2 subunits contribute to the permeation pathway, they do not contribute equally to the selectivity filter. Thus, swapping asparagine for glutamine (N → Q) in NR1 affects the Ca^2+^ permeability of the channel but not its Mg^2+^ block. The substitution in the TM2 region of the NR2 subunits affects the Mg^2+^ block, but not the Ca^2+^ permeability. Verdoorn et al. (1991) and Rozov et al. (2012) proposed that the amino acid occupying the Q/R site of the AMPA receptor subunits is controlled by RNA editing and determines the conductance, rectification and divalent cation permeability of the ion channel. Interestingly, at least the Kainate receptors in some brain areas are also inhibited by ethanol, which is suggested to affect the formation of neural circuit during brain development and the anxiolytic effects on ethanol i.e., Fetal alcohol spectrum disorders (Carta et al., 2003; Moykkynen and Korpi, 2012).

### Functions of NMDA receptors

- A unique property of the NMDA receptor is its voltage dependent activation, a result of the ion channel block by extracellular Mg^2+^ ions.
- The voltage dependent flow of Na^+^ and Ca^2+^ ions into the NMDA receptor, a glutamate receptor is the predominant molecular device for controlling synaptic plasticity.
- Calcium flux through the NMDA receptor is thought to play a critical role in pre- and post-synaptic plasticity, a cellular mechanism for learning and memory.
- NR1/NR2C and NR1/NR2D complexes possess much weaker Mg^2+^ blocking action than the NR1/NR2A and NR1/NR2B NMDAR complexes, indicating that sensitivity to the endogenous voltage blocker differs directly according to the subunits involved (Loeches and Guerri, 2011).
- The NMDA receptor plays an essential role in brain plasticity that is, the ability of the brain to change in response to external stimuli, such as during learning and memory.
- The NMDA receptor has been shown to play an essential role in both the strengthening of synapses, neuronal differentiation, through long-term potentiation (LTP), and the weakening of synapses, through long-term depression (LTD) and is implicated in the memory and special learning function (Barria and Malinow, 2002, 2005).
- AMPARs play a role also in modulation of the strength of neurotransmission as they can serve channels for the entry of calcium, an important second messenger (Huettner, 2003; Moykkynen and Korpi, 2012).

## NMDA receptor splicing and alternative splicing mechanism

The nearly transcribed precursor mRNA (pre-mRNA) contains non-coding sequences called introns, which must be removed before being transported to the cytoplasm and translated. The removal of insertions is carried out by a process termed splicing. Molecular modification such as splicing or RNA editing can alter Glu receptor properties. Most exon are constitutive, meaning that they are always included in the final mRNA. Regulated or alternatively spliced exons are sometimes included and sometimes excluded from the mRNA. These exon can also be termed “cassette” exons. To generate multiple transcripts from one gene, the splicing machinery can alter exon inclusion in many ways. The most common is the alternative inclusion of a cassette exon or the retention of an intron. When a gene has multiple cassette exons, they can be mutually exclusive; the final mRNA includes only one. Exon size can also be changed by the use of an alternative splicing site. Alternative splicing is not exclusive to the coding region of the mRNA. Some genes contain multiple promoters (5′ end of the mRNA) and polyadenylation sites that can be alternatively included in a given mRNA. Studies of the molecular basis of splicing revealed the existence of exonic and intronic *cis*-acting regulatory sequences that bind trans-acting factors and thus influence splice-site selection. These *cis*-acting elements are relatively short, usually 4–18 nucleotides, and are classified as exonic or intronic splicing enhancers or silencers. The regulation of alternative splicing is complex, since predicted sequences that identify an inclusion/exclusion of the exon are highly degenerate and occurs at high frequency in the genome. Furthermore, exception to the “rules” of splicing exist in abundance (Yu et al., 2008).

### NR1 subunits

**Figure 5:**
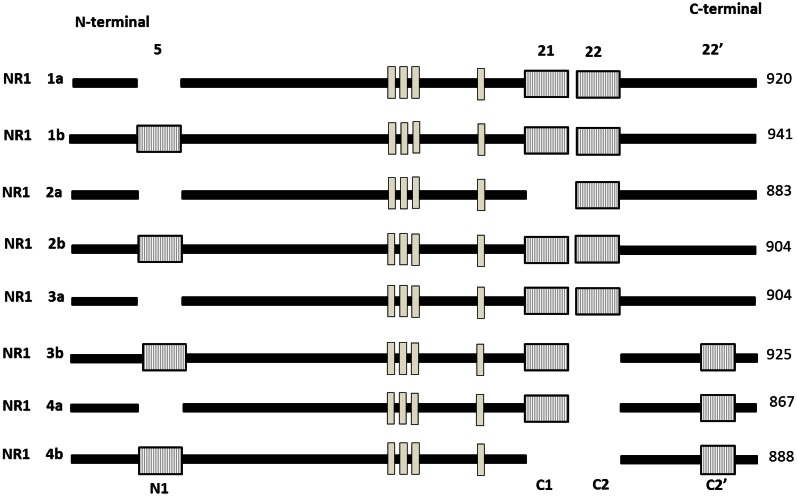
**Schematic representation of modular exon structure of the eight functional NR1 splice variants.** Absence of (1–4A) or presence of (1–4B) of a 21 amino acid sequence close to the N-terminal region also illustrated. The length of the mature protein is given in amino acid (aa) in right side. There are three different deletions at the C-terminal end. Exon 5 (63 bp), exon 21 (111 bp) featuring an ER retention signal, exon 22 (356 bp), 22′ alternative C-terminal (66 bp) featuring a divalent motif for enhanced ER export. Trans membrane domain (TM 1–4) are shown in white box. [Adapted and modified from Hynd et al. (2004)].

Gene expression is regulated not only at the level of transcription but also through the alternative splicing of pre-mRNA. Previous studies showed that ethanol attenuates NMDA receptors function in dose-related fashion *in vitro* (Kumari et al., 2003; Anji and Kumari, 2006, 2011). The ubiquitously expressed NR1 is the only NMDAR subunit encoded by only one gene. NR1 exists as eight different splice variants due to the alternative splicing of exon 5 (NR1) and exon 21 and 22 (C and C2). Figure 5 shows that the eight splice variants exist as combinations of a two splice form involving the presence or absence of the NR1 cassette and a four splice form involving the combination of the C-terminal cassette (Bradley et al., 2006; Horak and Wenthold, 2009; Ferreira et al., 2011). These splice variants exhibit heterogeneity with respect to agonist and antagonist affinity, zinc modulation, and regional and developmental expression pattern. It has also been shown that the splicing of exon 5 can influence the deactivation properties of NMDAR (Rumbaugh et al., 2000). NR1 splicing can also affect intercellular trafficking, colocalization and anchoring of signaling pathways to the receptors complex (Standley et al., 2000). The C1 cassette features an ER retention motif impeding surface expression of NR1-1 and NR-3 isoforms. Two other exons shown to be regulated in response to activity are the stress axis-regulated exon (STREX) in the BK potassium channel mRNA, which changes the firing properties of the channel and an exon in the *Apoliporotein* E receptor 2 mRNA (Apoer2), which is required for Reelin-depedent enhancement of Long-term potentiation (LTP). For NR1–3 variants, however, lower export efficiency might be compensated by the presence of a PDZ binding motif in the C2′ cassette and the subsequent interaction with PDZ proteins (Cavara et al., 2009). As neither NR1–2 nor NR1–4 contains the C1 cassette or features the retention signal, and in addition, NR1–4 has the C2′ cassette PDZ interacting motif (Xia et al., 2001). Bradley (2006) have demonstrated that the removal of C0 and C1 domains from the NR1 subunit is required for downstream signaling to the CRE-dependent gene expression. Although the C1 cassette also has protein kinase C (PKC) phosphorylation sites, and phosphorylation affects the subcellular distribution of NR1 (Ehlers et al., 1995). Interestingly when all eight NR1 isoforms are co-expressed in various combinations with one of the four NR2 subtypes in human embryonic kidney 293 cells, the sensitivity depends on the combination of NR1-3b/NR2C, NR1-3b/NR2D, and NR1-4b/NR2C pairs, which are most weakly inhibited by ethanol, and the NR1-2b/NR2C pair which is most strongly inhibited by ethanol (Acosta et al., 2010; Flores-soto et al., 2012). Their findings provide important clues regarding the contribution of NR1subunits, and their alternative splicing mechanism leads for physiological and pathophysiological conditions. These splice variants vary considerably in their properties and are differentially localized in adult and developing animals.

### NR2 subunits

The four NR2 subunits (NR2 A–D) are independent transcripts from our genes and they are expressed developmentally differently and region specifically. NR2D two splice variants are known to differ in their C-terminal (Ishii et al., 1993). Expression patterns of NR2 subunits in the brain are regulated developmentally (Del Valle-Pinero et al., 2007). NR2B and NR2D are expressed prenatally, whereas NR2A, 2C mRNA are first detected around birth (Table 1). As the expression of NR2B and NR2D decline, the mRNA levels of NR2A and NR2C increase concomitantly. The most prominent development change is NR2B mRNAs, which are abundantly expressed in the cerebellar granules cells on embryonic day 7 through the first post-natal week, then almost disappear by P14 being replaced by NR2C mRNAs (Watanabe et al., 1994; Bendova et al., 2009). The NR2 subunits show significant difference regarding their electrophysiological properties and are largely responsible for heterogeneity of NMDARs *in vivo*. The main difference between the NR2 subunits concerns the voltage-dependent block by Mg^2+^ ions, the translation of the ligand into a pore opening (gating) and Ca^2+^ permeability.

**Table 1:** **NMDA receptor subunits (NR1,NR2 and NR3) of mRNA distribution and their functions. (Adapted from Benarroch, 2011)**.

**Subunit type**	**NR1 (GluN1)**	**NR2 (GluN2)**	**NR3 (GluN3)**
Predominant distribution	Throughput the CNS	Hippocampus (NR2A, NR2B)	Neurons
		Neocortex (NR2A, NR2B)	Oligondedrocytes
		Cerebellum in purkinje cell (NR2A, NR2C), molecular (NR2B) and granule cell (NR2C) layers	
		Cortical interneuron (NR2C)	
		Brainstem (NR2C)	
Variants	Eight splice variants		
Function in NMDAR	Obligatory	NR2A or NR2B are most common in NR1/NR1 receptors	Activated by binding or glycine only
	Binding glycine	Binding glutamate	
Binding site	Glycine	Glutamate	Glycine
Modulatory sites	Polyamines	Zn^2+^	–
	Protons	Redox site	
		Nitric oxide site (nitrosylation)	
Predominant location	Postsynaptic density (PSD) and extra synaptic	NR2A; PSD	Dendritic spines during development
		NR2B: Extrasynaptic (NR2A/NR2B ratio increases in adult brain)	Oligodendrocytes

#### NR2B subunit splice variant

The NR2 subunit's gene undergoes alternative splicing. The proto-typical mouse NR2B gene has three non-coding exons at the 5′UTR and the ATG is loaded in exon 4 (exon 1′, 1, 2, 3) (Tabish and Ticku, 2004). The variants are the result of an alternative exclusion of exon 2 alone or in combination with different length deletion of exon 1 (Bendova et al., 2009). The remaining two variants result from the use of alternative acceptor site in exon 2 and the exclusion of exon 3. NR2B exon two contains many regulatory sequences, including binding a site for the transcriptional factor Sp1 and a cyclic AMP (cAMP) responsive element (CRE) site. Although gene transcription can proceed without exon 2, these genetic elements are involved in the regulation of ethanol-induced changes and NR2B expression (Mei and Maharaj, 2005). This indicates a role of exon 2 splicing in the ethanol responsive regulation of NR2B gene expression.

### NR3 subunits

In recent studies, widespread distribution of these subunits has been suggested, with NR3B found in the cortex, cerebellum, hippocampus, striatum, sub-stantianigra and spinal cord of an adult rat (Wee et al., 2008) (Table 1). Furthermore, 900 aa (ending in an arginine) has been reported for mouse and human NR3B, while all other studies consistently give the length of the mature proteins as 1003 aa with a serine at position 900. Considering the fact that one exon/intron boundary is located between position 899 and 900 of a mouse NR3B and it is entirely considerable that alternative splicing at this site generates an alternative splicing of the C-terminal region (Nishi et al., 2001). Regarding the amino acid sequence of NMDA receptor subunits, a mouse and human NR3 share a 74.9% sequence identity. NR3 subunits contain many of the typical NMDA receptor structural features, in particular the motif SYTANLAA, which is conserved in all ionotropic glutamate receptors. These motifs appear to be important to regulation of NMDA receptor channel gating (Chang and Kuo, 2008). It is important to note that one potentially critical difference between NR3 and the other NMDA receptor subunits is an alteration in the “Q/R/N” site, which is critical for high Ca^2+^ permeability of the NMDA receptor. The “Q/R/N” site is located inside M2 (pore-forming region) and in NR3 subunits the asparagine is changed to glycine (Traynelis et al., 2010). NR3 might depend on the other subunits present in a cell, either assembling into tri-heteromers with NR1 and NR2, or recruiting only NR1 into excitatory glycine receptors. But still a study is needed to link the NR3 subunits functional determination to specific binding elements.

Hence, polymorphisms without amino acid alteration in multiple ethanol sensitive candidate genes probably increase the risk of alcoholism by modulating the expression of alternative spliced variants. Still a few question need to be clarified, such as how ethanol affects the splicing machinery and alcoholism-associated polymorhphisms. The oxidative stress induced in ethanol metabolism might be a factor; ethanol consumption result in the production of reactive oxygen species (ROS) by the mitochondrial electron transport chain, cytochrome P450 and activated phagocytes and chemical stresses are known to affect the splicing of specific pre-mRNA. The importance of splice-site enhancers becomes apparent when they are changed by mutation, which can alter their interaction with trans-acting factors. It remains to be determined whether all missence mutations cause a pathological state by an amino acid exchange or are actually unrecognized splicing mutations. I hypothesize splice-site selection is regulated by extracellular signals, the analysis of these signal transduction pathways might provide new insights and novel chances for therapeutic approaches.

## Ethanol and NMDA receptors

**Figure 6:**
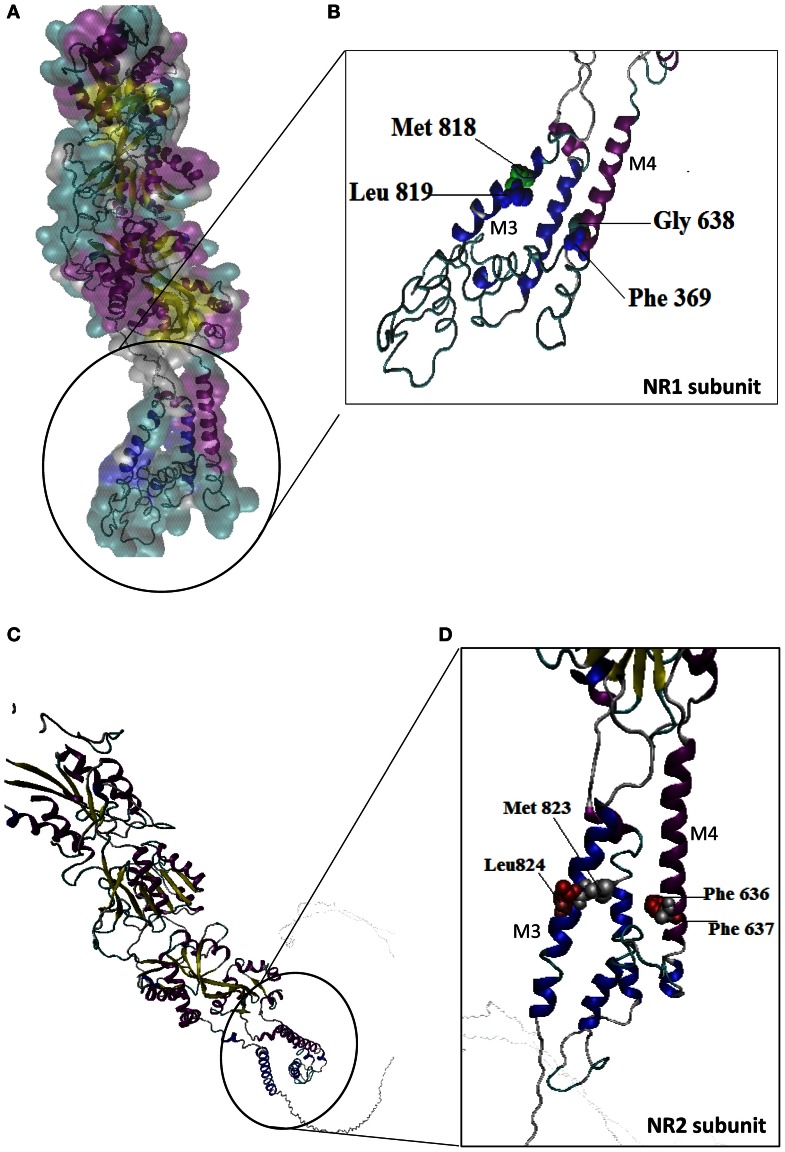
**Molecular structure of the NR1/NR2 subunit pairs shows a continuous molecular view.** A model of the mouse NR1/NR2 subunit was derived by homology modeling and is depicted as a stereo view transparent grid surface ribbon model **(A,C)**. Position in NR1 subunit was highlighted green (Met818, Leu819) and blue (Gly638, Phe639) residue are possible binding region of M3 and M4 domain. **(B)** Position in NR2 subunit M3, M4 domain interact with ethanol sensitivity are illustrated in red (Met823/Leu824) and silver color (Phe636/Phe637) binding site **(D)**. NR1 (Accession no. NM_01177657.1) and NR2 (Accession No. NM_008170.2) subunit 3D structure prediction by using PHYRE2 automatic (http://www.sbg.bio.ic.ac.uk/phyre2) server. Ethanol binding active residues marked according to Ren et al. (2012) by using PyMol program (www.pymol.org).

Alcohol drug addiction appears to have a cellular and molecular basis, and some of the most challenging questions in addiction research relate to understanding how the brain modifies its structure and function in response to alcohol exposure (Table 2). Signal transmission among nerve cells, or neurons is mediated primarily by neurotransmitter molecules that are released from the signal-emitting (i.e., presynaptic) cell and interact with specific molecules (i.e., receptors) on the surface of the signal-receiving (i.e., postsynaptic) cell. However, genes whose activity is altered in the presence of alcohol may either be contributing to alcoholism development or may be reacting to alcohol's presence. Dynamic regulation of the efficacy of excitatory synaptic transmission is critical in experience-dependent plasticity and the remodeling of neuronal connections. It is well-established that NMDAR is a major target of alcohol in the brain and has been implicated in ethanol-associated phenotypes such as tolerance, dependence, withdrawal, craving and relapse (Pignataro et al., 2009). Calcium influx through NMDA receptors is thought to play a major role in activity-dependent synaptic plasticity via calcium-regulated signaling events in the dendritic spine. The entry of calcium into the post-synpase via the NMDA receptor permits the coupling of electrical synaptic activity to biochemical signaling via activation of Ca^2+^ -dependent enzymes and downstream signaling pathways. In this way, calcium influx through the NMDA receptor can lead to long-term changes in synaptic strength and other cellular modifications, including alternations in synaptic structure or connectivity. The inhibitory actions of ethanol on the activity of the channel were demonstrated by measuring NMDA receptor excitatory postsynaptic potentials/currents (EPSPs/EPSCs) in slices from many brain regions such as the hippocampus (Kolb et al., 2005), cortex, (Yaka et al., 2003), amygdala (Calton et al., 1999), nucleus accumbens (Maldev et al., 2002) and dorsal striatum (Yin et al., 2007). There are several studies demonstrating that the maximal inhibition of channel activity in the presence of ethanol was observed in NR1-2B/NR2C, while the minimal one was found in NR1-3B/NR2C, NR-3B/NR2D, and NR1-4B/NR2C. These findings suggest that the overall sensitivity of an individual NMDAR to ethanol depends on specific combinations of NR1 and NR2 subunits. NR1 is an essential component found in all tetramers, while, different NR2 members are incorporated based on age and nervous system region. In a previous study, the Ronald et al. (2001) and Ren et al. (2012) laboratory demonstrated that the exact binding sites of the ethanol within a methionine residue (Met818), leucine (Leu819) and a similar glycine and phelanine residue (Gly638, Phe369) in the third/fourth membrane-associated (M3) and (M4) domains of the NMDA receptor NR1 subunit influenced an apparent affinity with alcohol molecules (Figure 6), whereas the NR2A subunit, has specific residues Met823, Leu824 and Phe636, Phe637 and a strong ethanol binding sites with domain M3 and M4, respectively. Taken together, the structural model predicts the presence of four site of alcohol action on the NMDA receptor, each containing four pairs of positions in the NR1/NR2 subunit: Gly638/Met823, Phe639/Leu824, Met818/Phe636 and Leu819/Phe637 (Figures 6A–D) and these results suggest that there are amino acids within the third and fourth transmembrane domain interfaces.

**Table 2:** **Glutamatergic hypothesis of alcoholic brain injury (Adapted from Tsai and Coyle, 1998)**.

**Clinical presentations**	**Ethanol's glutamatergic effects**
Euphoria and dependence	Increased mesoaccumbenal dopamine neurotransmission. Upregulation of glutamate receptors.
Blackout	Impaired long-term potentiation. Acute attenuation of NMDA receptor neurotransmission on context of chronic up-regulation of NMDA receptor.
Wernicke-Korsakoff syndrome, cerebellar degeneration and cerebral atrophy	NMDA receptor supersensitvity leading to excitotoxicity. Decreased magnesium, zinc, thamine. Increased nitrous oxide production, hypercortisolemia.
Fetal alcohol syndrome	Block of NMDA receptor's trophic effects.
	Decreased glutamate receptors density postnatally.

However, between 3 and 5 h after commencement of detoxification from ethanol, there is an enormous increase in glutamate release, both NR1 and NR2B subunit's polypeptide levels being increased in numbers but decreased in sensitivity during the chronic alcoholization stage (Kumari and Anji, 2005). Interestingly, various studies have found that there may be a role of CREB and its target genes, such as the neuroopeptide Y (NPY), the brain-derived neurotropic factor (BDNF), the activity-regulated cytoskeleton-associated (Arc) protein, and the corticotrophin-releasing factor (CRF) in the development of alcoholism (Moonat et al., 2010). More recently, further analysis was done to identify the molecular mechanism underlying the increase in the expression of NR1 subunits, and it was found that there was an enhancement of NR1 mRNA stability upon chronic exposure to ethanol, possibly via an association with the RNA binding protein GII β (Anji and Kumari, 2011). The presence of a cis-acting region in the 3′UTR of the NR1 mRNA and its interaction with three trans-acting proteins (GIIβ subunits of alpha glucosidase II, and annexin-II) expressed in cultured FCN exposed to chronic ethanol was identified. From the result, it was suggested that the GIIβ and other NR1 mRNA binding trans-acting proteins modulate mRNA stabilization and translation of NR1 mRNA. Additional work will be needed to detail characterization of these proteins and a peptipedomic approach may lead to unexpected functions.

## Ethanol effect of brain shrinkage

Studies in experimental animal models have identified several common anatomical abnormalities that serve to highlight brain region, areas, and epochs that are vulnerable to the effects of ethanol during development (Gabriel et al., 2002; Qiang et al., 2002). Application of the MRI (magnetic resonance imaging) in human alcohol studies provides the unique opportunity of *in vivo* examination of white matter microstructural degradation reported in neuropathological study and possible previously only on post-morterm examination (Jansen and Pakkenberg, 1993; Sowell et al., 2001). Most of these studies have found small but usually statistically significant differences in the brain volumes of man alcoholics compared with those of non-alcoholics (Table 2). Although it is not clear exactly how alcoholism leads to a reduction in brain weight and volume, it is clear that this does occur during active alcohol abuse, and that some recovery of brain mass does occur during abstinence. Early studies were confirmed by using computerized tomography (CT) brain shrinkage (Kril and Halliday, 1999), a common marker of brain damage, and pneumoencephalography (neuroradiological techniques). Later on by measuring the increase in the cerebrospinal fluid (CSF) surrounding the brain which is an indication of the size of the lateral ventricle (a CSF- filled cavity inside the brain that increase in size as the brain shrinkage), based on clinical and radiological studies on an experimental model, some alcohol-related damage appears to be reversible. Thus, cortical and hippocampal neurons that contain the peptide vasopressin may be sensitive to chronic ethanol-induced neurotoxicity in both rats and humans (Harding et al., 2000).

Currently various techniques used to detect abnormalities of brain-damage with respect to alcohol, such as magnetic resonance spectroscopy-MRS, (Van der Graaf, 2010), DTI (diffusion tensor imaging for white-matter microstructure) permit quantification of the directionality, and coherence of white matter fiber tracts and are based on the measurement of self-diffusion of water molecules (Pfefferbaum and Sullivan, 2005), PET (positron emission tomography) allows the measure of *in vivo* μ-opiate receptor availability with carfentanil positron emission tomography, SPECT (single photo emission computed tomography measuring regional cerebral blood flow rCBF) techniques allows the assessment of the parameters of tissue function regionally, quantitatively, and non-invasively. Taken together, the data demonstrate a selective neuronal loss, dendritic simplification, and reduction of synaptic complexity in specific brain regions of alcoholics. It remains uncertain how these cellular lesions relate to selective loss of white matter that appears to occur particularly in frontal lobes. One reason these frontal lobe changes are more evident is the greater proportion of white matter to cortical gray matter in the frontal regions. Frontal lobe shrinkage has been reported with or without seizures, with recent studies suggesting that temporal lobe shrinkage occurs particularly in individuals with alcohol withdrawal seizure history. Jagannathan et al. (1996) describe the decreases in the amounts of N-acetyl aspartate in the frontal lobe, and a measure of neuron levels, also illustrate frontal lobe degeneration in alcoholics. In an extension of these findings, recently Hayes et al. (2013) demonstrate 24 h ethanol exposure is enough to elicit signs of alcohol-induced brain damage in adult male Sprague–Dawley rats. Further, reactive gliosis may be a more sensitive marker of alcohol-induced damage in the hippocampus region. Alcoholics with more severe brain disorders, such as Wernike's and/or Korsakoff's syndrome, show a more significant reduction in white matter and a more extensive brain region degeneration, which is consistent with the greater alcohol consumption associated with more severely damaged individuals. But, our understanding of the nature of the frontal lobe together with its associated white gray matters appears to be the most sensitive to alcohol-induced damage. Over the past decade, the use of neuroimaging technology has proven to be a valuable tool in understanding alcohol addictive disorders and can provide a bridge between preclinical and clinical work.

## Ethanol effect of growth associated protein (Gap-43)

Cellular mechanisms by which alcohol affects the migration of immature neurons in the developing brain have not been resolved, despite more than two decades of research. The neurogenic deficits only become apparent when the adult fetal alcohol exposed mouse is behaviorally challenged. The growth associated protein of 43 kDa (also known as B-50, F1, or neuromodulin, Gap-43), which demonstrates a spatial and temporal pattern of expression during development, plays an important role in the development of the nervous system, particularly in the determination of neuronal cell lineage and in neurite differentiation, axonal growth, extension, and synaptogenesis secretion of both catechoalmine and neuropeptides (Strittmatter et al., 1995; Mani et al., 2000). However, up-regulation of the Gap-43 protein and/or mRNA is highest in the hippocampus in the first postnatal week and then decrease by 90% over the next few weeks (Bolognani et al., 2007). Expression of GAP-43 is also developmentally regulated in the cerebellum with similar reductions in levels of this mRNA in maturing cerebellar granule cells. The developmental decrease of GAP-43 gene expression from adolescence to adulthood suggests a role of this protein in the maturation of both the hippocampus and cerebellum, which could be affected by binge alcohol consumption. Interestingly, the changes were region specific, with the cerebellum showing decreased expression of GAP-43 and BDNF and the hippocampus increased expression of these proteins. These results suggest that alcohol affects brain function during adolescence in part by interfering with the normal remodeling of synaptic connections characteristic of this developmental period. Recently Yanni and Lindsley (2000) reported that ethanol inhibits development of dendrites and synapses in rat hippocampal pyramidal neuron cultures; 6 days of ethanol treated (200, 400, or 600 mg/dl) to the medium, beginning at the time of plating, resulted in decreases in total dendritic length per cell, dendrite, number per cell, length of individual dendrites, and synapse number per innervated dendrite but had no effect on cell survival. These studies suggest that younger rodent or human brains have a heightened vulnerability to alcohol-induced effects. However, very little report shows that ethanol effects reduction of axonal growth and abnormalities in dendrites. For example, a recent study showed that single acute ethanol exposure significantly decreased the level of Gap-43 and protein in the cerebellum but increased the level of mRNA and protein in the hippocampus (Kulkarny et al., 2011).

## Ethanol effect on interference with signaling by neurotrophic factors

Considerable research efforts are necessary to ascertain how the various signaling pathway are altered by alcohol when administered either chronically or intermittently, on the intercellular signaling. This is also true for the C-terminal region is that involved in NMDA receptor biogenesis and subcellular targeting (Standley et al., 2000). The C-terminal region is also involved in linking intracellular signaling pathways to NMDA receptors by harboring (1) kinase phosphorylation sites (Tingley et al., 1997); (2) binding domains for cytoskeletal proteins (Ehlers et al., 1995); (3) interaction sites for actin-binding proteins (Shen et al., 2000); and (4) binding motifs for many signaling molecules (Muller et al., 1996). NMDAR subunits phosphorylation is critical for activity dependent regulation of NMDAR trafficking and function. On the other hand a toxic effect of alcohol on endocytosis could affect several important neuronal activities, which depend on the endocytic process (Rab5, Rab11, Ap-2/clatherin coat), including synaptic vesicle recycling (SNX9 or RhoA, Arf6, Cdc42, EEA1), regulation of number of signaling receptors, growth cone navigation, and neuronal migration and hippocampal plasticity.

**Figure 7:**
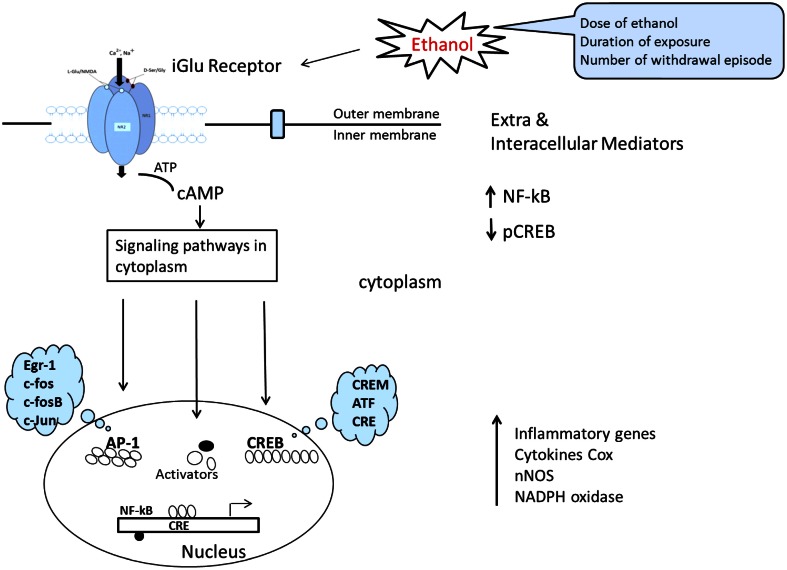
**Schematic representation of signal transduction route where by ligands at the cell surface interact with, thereby activate, membrane NMDAR and result in altered gene expression.** This converts ATP to cAMP, the convergence of comprehensive signaling transduction in cytoplasm after ethanol influence is directed toward a reduction in CREB signaling and an increase in NF-κB signaling. Finally CREB is able to regulate the transcription of downstream target genes and leads to imbalance between procytokine-oxidative stress and pro-survival gene transcription. [The model was adapted and modified from Johnston (2003) and Cao et al. (2012)].

The mechanisms of ethanol-induced cerebellum granules' cell deaths are complex and likely reflect the combined outcomes of promoting intrinsic apoptotic pathways and inhibiting anti-apoptotic signaling. Multiple mechanisms may interplay: these include inhibition of NMDA receptors, interference with signaling by neurotrophic factors, induction of oxidative stress, and modulation of retinoid acid signaling, disturbance of potassium channel currents, thiamine deficiency and disruption of translational regulation. Despite thousands of published studies on alcohol cytotoxicity, the true mechanism of alcohol-induced neuronal damage remains unclear. Some investigators suggest that cerebellar granules' cell losses may occur as a delayed reaction secondary to the loss of purkinje cells, other evidence indicates that ethanol may cause death in granules' cells by the increases of caspase-2, -3,-6,-8 and, -9 activities, DNA fragmentation and mitochondrial permeability. Ethanol inhibition of the anti-apoptotic effect of NMDA is associated with a change in the properties of the NMDA receptors. This is indicated by decreased ligand binding, decreased expression of NMDA receptors' subunit proteins, and decreased functional responses, including stimulation of increases in interacellular Ca^2+^ and induction of brain-derived neurotrophic factor (BDNF) expression (Bhave et al., 1999). Many of the dramatic alternations described in exogenous NMDA can counteract ethanol-induced apoptosis of cerebellar granule cells and offer neuro-protection. NMDA's protection against ethanol-induced cell death is believed to mediated by a mechanism that involves the nNOS-mediated signaling pathway (NO, cGMP, and PKG). Further studies demonstrate that the ethanol inhibits DBNF mediated activation of PI3K/Akt and JNKs and blocks BDNF-stimulated AP-1 activation (Figure 7). More recently, scientists have become increasingly aware of the potent action of oxidative stress, which has been proposed as a potential mechanism of ethanol-induced neuro-degeneration in the developing, mature, and aging cerebellum. In short, the evidence, both pro and con, regarding prenatal ethanol exposure also delays the migration of cerebellar granules cells during the postnatal period, indicating that prenatal exposure, to ethanol is sufficient to disrupt the neuronal migration program and their permanent consequences.

## Alchol-induced changes in oxidative stress

Acute and chronic alcohol exposure leads to a high concentration of unsaturated fatty acid, high oxygen consumption rate, low anti-oxidant content, and high content of metal catalyzing free radical formation in the liver. Neuronal function and survival relies on a constant supply of oxygen and glucose to produce ATP through glycolysis and mitochondrial respiration. Although elevations in extracellular glutamate play a vital role in mediating both ischemic and hypoglycemia, energy deficiency results in the dysfunction of the presynaptic neurotransmitter release and leads to a net increase in extracellular glutamate, causing neurotoxicity [(1) increased production of toxic ROS such as nitric oxide (NO, NOS), superoxide (O^−^_2_), hydrogen peroxide, (2) alternation in the organization of the cytoskeleton, (3) activation of genetic signals leading to cell death (apoptosis), and (4) mitochondrial dysfunction]. The major pathway of the oxidative metabolism of ethanol in the brain tissue involves the production of acetyaldehyde by cytosolic alcohol dehydrogenase (ADH). Thus, regarding the NAD^+^/NADH ratio, alcohol metabolism may result in gene activation, and leads to alter many important cellular reactions and the relative quantities of NAD^+^ to NADH^+^ fluctuate in response to changes in metabolism (Flora et al., 2012; Yeligar et al., 2012). Qin and Crews (2012) suggest that chronic ethanol induces brain NADPH oxidase gp91^phox^ (NOX2) up-regulation and neurodegeneration in adult C57BL/6 mice that mimics findings in the human alcoholic brain.

It is likely that alcohol-induced acetylation is required for the activation of alcohol-metabolizing enzymes, which induced oxidative stress. This, in turn, induces acetylation that creates an amplifying “autocrine loop” between alcohol metabolism and epigenetic events. Emerging investigations have provided evidence that hydroxyl radicals generated by oxidative stress interfere with the ability of DNA to function as a substrate for DNMT, which result in global hypomethylation (Esfandiari et al., 2010). Furthers studies must unravel the exact composition and molecular dynamics of the NAD^+^/NADH (are their brain-specific or ethanol-specific differences?), its regulation by mitochondrial (what interacts with what via which domain and under which precise conditions of opening/closure?) and extra mitochondrial effectors and its pharmacological manipulation.

## Ethanol effect on transcriptional regulation

### Epigenetics regulation

**Figure 8:**
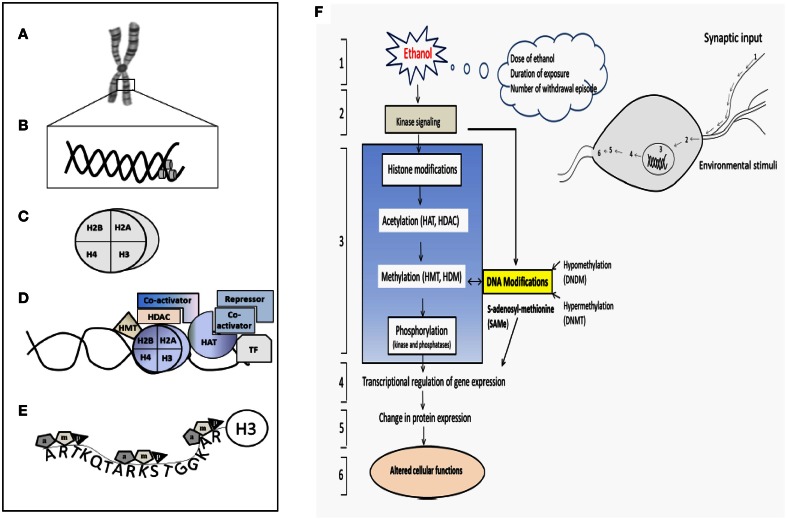
**Schematic representation of the epigenetic modification and a hypothetical complex transcription. (A)** Chromosome, **(B)** Nucleosome (DNA), **(C)** Histone, **(D)** Different components involving enzymes, co-activator, repressor and transcription factors essential for the histone modification. **(E)** H3 histone tail and their major component of epigenetic factors acetylation (a), methylation (m), and phosphorylation (p). **(F)** Schematic diagram shows pathway involved in the ethanol-induced epigenetic changes and role of methylation and leading to the expression of genes and proteins. (1) Ethanol exposure of nerve cells (environmental stimuli), (2) these external stimuli result in changes in downstream genes and kinase signaling pathway. (3) Histone Modification (Acetylation, methylation and phosphorylation) and their responsible enzymes. (4) Transcriptional regulation and gene expression, (5) translation (protein expression), (6) altered cellular function. HAT- histrone acetyl transferase; HDAC- histone deacetylase; HDM-histone demethylase; HMT-histone methyl transferase. The model was adapted and modified from Shukla et al. (2008).

Only recently, more insight is gained in the alcoholism and alcohol related diseases are the result of the complex interaction of multiple genetic and environmental factors. The dynamic multi-level interactions between genetic and environmental components are responsible for the heterogeneity and complexity of alcohol-induced diseases (Brooks and Lipsky, 2000; Putignano et al., 2007; Roth and Sweatt, 2009; Starkman et al., 2012). New evidence shows that numerous type of epigenetic modification on both DNA and nucleosomes (Figures 8A,B), including methylation and acetylation, which could affect gene regulation and expression. Furthermore, there are histone variants of different types e.g., H3.1, H3.2, and H3.3; H2A1-6, H2A.7. Covalent histone modifications appear to act sequentially or in combination to form a recognizable code that is identified by a specific protein to regulate distinct downstream events such as transcriptional activation or repression (Figures 8C–E). Histones are subject to various post-translational modifications such as (1) acetylation, (2) methylation, (3) phosphorylation, (4) ubiquitinylation, (5) ADP-ribosylation, and (6) sumoylation, all having an impact on gene transcription (Figure 8F).

Clearly, progress has been made in linking the alcohol exposure at crucial developmental stages could result in alterations of the epigenetic marks, thus altering the expression of key genes resulting in physiological and or behavioral changes; not only in the exposed individuals, but also in their offspring (Fagiolini et al., 2009). This initiative is designed to promote on innovative hypothesis and exploratory research to determine if and how alcohol exposure changes the epigenetic patterns in both the exposed individuals and their offspring, and to determine if alcohol-induced oxidative stress changes the patterns, of gene expression and causes brain injury (Table 2). Since ethanol has been found to alter DNA methylation in the brain, liver, esophagus, colon, uterus, and testes, it is important to identify the genomic regions that are likely targets for alcohol-induced CpG methylation changes (Ashley et al., 1993; Ravindran and Ticku, 2005; Pignataro et al., 2009; Hillemacher, 2011). The unmethylated CpG islands are associated with transcriptionally active promoters; how CpG islands remain unmethylated is still unclear. Stress, including that resulting from dietary methionine/choline, folate, zinc, and other micronutrient insufficiencies, as well as excessive alcohol intake, can lead to global DNA hypo methylation (Table 3). Alternation in normal gene silencing or activation results in inappropriate gene transcription, leading to tissue dysfunction and disease. The major pathway of the oxidative metabolism of ethanol in the brain involves the production of acetaldehyde by (ADH) cytosolic alcohol dehydrogenase (Bleich et al., 2006). As mentioned previously, an oxidation is accompanied by the reduction of NAD^+^ to NADH, thus, altering the cellular redox state by decreasing the NAD^+^/NADH ratio. This imbalance may result in altered physiology and an impact on long-lasting alternation in gene expression.

**Table 3:** **Ethanol-induced histone and DNA modifications**.

**Type of modification**	**Enzymes**	**Target gene transcription**	**Ethanol effect**
**ACETYLATION**			
H3 K9	GNAT	Activating	Increase
H3 K14 or K18 or K23	MYST	Activating	Non-detected
H3 and H4	CBP/p300	Activating	Increase
**DEACETYLATION**			
H3 H4	HDACs1–11	Activating	Ethanol up-regulated genes
H3 K9	SUV39H1	Silencing	Ethanol down-regulated genes
**METHYLATION**			
H3 K4	Set1	Activating	Ethanol up-regulated genes
H3K36	Set2	Activating	Ethanol up-regulated genes
H3K79	DoT1L	Activating	Ethanol up-regulated genes
H3K27	EZH2	Silencing	Non-detected
H4K20	SUV4-20H1	Silencing	Non-detected
Methylated CpG	DNMT1, DNMT3a-3b	Decrease activity	Ethanol down-regulated genes
**PHOSPHORYLATION**			
H3 S10	RSK2	Not known	Increase
H3 S28	–	Not known	Increase

### Ethanol-induced histone modification

A currently available literature source showed that the epigenetic mechanisms have proven fruitful in numerous fields, including drug addiction. Here, I describe three major mechanisms of epigenetic regulation: DNA methylation, acetylation and phosphorylation, and summarize the major findings that have linked each of these mechanisms to drug addiction and their disorder (Tables 3, 4).

**Table 4:** **Epigenetic mechanism in neuronal disorders**.

**Disorder/Disease**	**Epigenetic modification**	**Organism and brain region**	**Mechanism and functions**	**References**
Synaptic plasticity	Histone acetylation	Rat, hippocampus	ERK/MAK activity leads to H3K14 hyper-acetylation	Levenson et al., 2004
		Rat, amygdala	TSA increases LTP	Yeh et al., 2004
		Rat, hippocampus	PKC activation enhances LTP and H3K14 acetylation	Miller et al., 2008
		Mouse, hippocampus	CBP deficiency result in H2B hypo acetylation	Alarcon et al., 2004
		Mouse, hippocampus	TSA enhances LTP and transcription of CRE	Vecsey et al., 2007
		Mouse, hippocampus	HDAC2 overexpression impairs, HDAC2 k.o	Guan et al., 2009
		Mouse, hippocampus	PcGprotein MII-deficiency enhances, TrxG protein and EED deficiency impairs LTP	Kim et al., 2007
	DNA methylation	Mouse, hippocampus	MBD1 deficiency leads to reduced LTP	Zhao et al., 2003
		Mouse, hippocampus	Inhibition of DNMT1 activity by 5-aza blocks LTP	Levenson et al., 2006
		Mouse, hippocampus	5-Aza and zebularine reduce mEPSCs and increase BDNF DNA methylation and transcription	Nelson et al., 2008
		Mouse, forebrain	Double knock-out DNMT1 and DNMT3a impairs Synaptic plasticity	Feng et al., 2010
Seizures	Histone acetylation	Rat, hippocampus	Pilocarpine-induced seizures leads to H4 hypo acetylation of GluR2	Huang et al., 2002
		Rat, hippocampus	Acute and chronic electroconvulsive seizures induce differential H3 and H4 acetylation	Tsankova et al., 2004
Schizophrenia	DNA methylation	Human post-mortem brain, cortex region	GAD67 and SOX10 promoter region are hyper methylated correlating with reduced mRNA level	Iwamoto et al., 2005; Guidotti et al., 2007; Huang and Akbarian, 2007
		Human post-mortem brain, frontal lobe	COMT promoter is hypo methylated correlating with increased mRNA levels	Abdolmaleky et al., 2006
ATRXS	DNA methylation	Human patient-derived cell lines	Mutations in ATRX result in aberrant DNA methylation	Gibbons et al., 2000
		Mouse, forebrain	ATRX form a silencing complex with MeCP2 that is disturbed in ATRXS	Kernohan et al., 2010
Rett syndrome	DNA methylation	Human lymphocytes mouse, hippocampus, cortex and cerebellum	MeCP2 mutations lead to reduced and CpG islands and acts a transcriptional repressor	Amir et al., 1999; Chen et al., 2001; Collins et al., 2004
	DNA methylation and histone acetylation	Mouse, cortex, cerebellum murine cells lines	MeCP2 mutations cause aberrant transcriptionally permissive	Shahbazian et al., 2002; Martinowich et al., 2003
			Histone acetylation and methylation marks	
Rubinstein Taybi Syndrome	Histone acetylation	Mouse, hippocampus	Reduced global HAT activity due to loss of function in CBP	Oike et al., 1999; Alarcon et al., 2004; Korzus et al., 2004
Fragile X mental retardation	DNA methylation	Human and mouse cell lines	Expansion of CGG or CCG repeats results in aberrant DNA methylation around FMR1 and FMR2 genes	Ashley et al., 1993; Chiurazzi et al., 1998
Alzheimer's	Histone acetylation	Human and mouse cell lines	APP intracellular domain acts as a Notch-like transcription factor associated with HAT TIP60	Herman et al., 2006; Haberland et al., 2009
Contextual memory Visual cortical plasticity	Histone acetylation/phosphorylation	Crab, central brain	Contextual training increased H3 acetylation	Federman et al., 2009
		Mouse, visual cortex region	Visual stimulation increases H3 phosphoacetylation via ERK/MAPK	Putignano et al., 2007
Object memory	Histone acetylation	Mouse, hippocampus, forebrain	CBP and p300 deficient mouse have impaired long-term object memory	Alarcon et al., 2004
	Histone acetylation/methylation	Muse, hippocampus	Estradiol enhances object memory and increases H3K14 but not H4acetylation	Oliveira et al., 2007; Zhao et al., 2010
			Nuclear inhibition of PP1 increases object memory as well as	
		Mouse, hippocampus	H3K14/H4K5 acetylation, H3K36 trimethylation and H3S100 phosphorylation	Koshibu et al., 2009
Fear memory	Histone acetylation	Rat, hippocampus	ERK/MAPK activation increases contextual fear memory and H3	Chwang et al., 2006
	DNA methylation	Mouse, hippocampus	VPA and SB rescue and H3 and H4 hyperacetylation	Dash et al., 2009
		Mouse, hippocampus	CBP deficiency impairs contextual fear conditioning	Wood et al., 2005
		Rat, hippocampus	Contextual feat training increases BDNF expression and reduce its promoter methylation	Lubin et al., 2008
Spatial memory	Histone acetylation	Mouse, hippocampus	CBP deficiency impairs spatial memory accompanied by H2B hypo acetylation.	Alarcon et al., 2004; Korzus et al., 2004
	Acetylation/methylation/phosphorylation	Mouse, hippocampus	Nuclear inhibition of PP1 increases spatial memory as well as H3K13/H4K5 acetylation, H3K36 tri-methylation and H3S100 phosphorylation	Koshibu et al., 2009
Taste memory	Histone acetylation	Mouse, insular cortex	TSA increases H2A and H4 acetylation in brain areas important for taste memories	Swank and Sweatt, 2001

#### Methylation

The emerging role of the epigenetic regulation of the NMDA receptor gene expression by alcohol and DNA methylation have been discussed to play a role in the pathophysiology of several psychiatric disorders such as depression (Frieling et al., 2007; Hillemacher, 2011), schizophrenia (Bleich et al., 2006) and psychostimulant drug exposure. DNA methylation of the CpG island within gene promoters is generally associated with transcriptional repression (Sharma et al., 2010). DNA methylation represses transcription, in part through the recruitment of the methylated DNA-binding protein, in particular methylated-CpG-binding protein 2 (MeCP2), MBD1-4 (Shahbazian et al., 2002; Martinowich et al., 2003). In the majority of cases, a higher methylation of the genomic sequence leads to an inactivation of the referring gene, while less methylation leads to activation (Egger et al., 2004). These studies identify that MeCP2 may have a special role in the brain and mediator between those two epigenetic phenomenoa- methylation and acetylation (Rodenhister and Mann, 2006). For example, in the Liang et al. (2003) studies have shown that the expression of alpha synuclein is enhanced in different brain areas of rats, while alcohol preference is inbred. An increased expression of alpha synuclein mRNA has been described in alcohol-dependent patients, with a correlation to obsessive alcohol craving (Bonsch et al., 2004). Rats fed an intragastic alcohol diet for 9 weeks exhibited decreased methionine, SAMe, glutathione and loss of DNA methylation by 40% (Qiang et al., 2010). This DNA hypomethylation can lead to an alternation in gene expression and chromatin structure resulting in increased DNA damage and strand breaks (Gibbons, 2006; Rani and Ticku, 2006; Pignataro et al., 2009), which predisposes cells to malignant degeneration. In a chronic alcohol exposed brain and liver, whether an interconnection exists between hyper-acetylation of H3K9, loss of methylation of H3K9, and increased methylation of H3K4, along with global hypo-methylation of DNA is under progress in the world (Figure 8F). Interestingly, another topic in the field of epigenetic alterations in alcohol induced changes is the methylation of the promotor region of HERP (homocysteine-induced endoplasmatic reticulum protein). HERP is an ER resident membrane protein, which regulates Ca^2+^ homeostasis and thus protects endothelial and neuronal cell integrity against oxidative stress. Furthermore, HERP mRNA expression was negatively correlated with its promoter methylation, but studies strongly suggest that suppressed expression of HERP under conditions of chronic alcohol consumption may be partially responsible for an elevated rate of seizures and other neurological disorders. Muschler et al. (2010) demonstrate that the DNA methylation status in the gene sequence of POMC (i.e., polypeptide pro-opiomelancortin) at single CpG sites differs between patients with alcohol dependence and health control, and have identified a specific cluster of the CpG island showing a significant association with alcohol craving.

There is considerable inter-individual variation in DNA methylation, which suggests that variation in CpG methylation might associate with variation in the nucleotide sequence. A genome-wide CpG methylation in the brain revealed a significant correlation between sequence polysmorphism and CpG methylation, most of which seemed to operate in *cis* (Qiang et al., 2010; Hillemacher, 2011). The promoters of the genes that encode the NR1, NR2B, NR2C, GluR1, GluR2, and KA2 subunits share several characteristics that include multiple transcriptional start sites within in CpG island, lack of TATA and CAAT boxes, and neuronal-selective expression. In most cases, the promoter region includes over lapping Sp1 and CSG motifs near the major initiation sites, and silencer elements, to guide expression in neurons (Myers et al., 1999). More recently, an animal study showed that chronic alcohol consumption in mice leads to a hypomethylation of specific areas in the genomic sequence of the NR2B, leading to a receptor up-regulation (Ravindran and Ticku, 2005). Similar investigation in human study showed a significant association between high life time drinking and high daily alcohol intake with lower DNA methylation of NR2B in alcohol-dependent patients undergoing alcohol withdrawal (Follesa and Ticku, 1995; Biermann et al., 2009). Taken together, these findings delineate the up-regulation of NR2B in alcohol dependence may be a modification of genomic DNA methylation. Additional studies identified a subsequent step in the signaling cascade induced by BDNF-Arc signaling and synaptic plasticity contributes to both dysphoria associated with a genetic vulnerability for anxiety and to anxiety induced by environmental stressors, such as alcohol withdrawal (Moonat et al., 2011). However, many trans factors (REST and CREB) have been found to directly interact with NR promoters to regulate with chromatin remodeling, which in trun play a role in the modulation of synaptic structure and function. But detailed direct evidence does not exist.

#### Acetylation and phosphorylation

Tremendous progress has been made in exploiting *in vitro* and *in vivo* studies to enhance that alcohol induces epigenetic modification in various organs i.e., spleen, liver, lung, testes, brain for post-translational modifications of the histone tail. Ethanol causes selective acetylation of histone H3 at Lys9 in primary cultures of rat hepatocytes, and increases histone acetyl transferase activity (Park et al., 2005). The status of histone acetylation depends on the activity of HAT and HDAC. In some instances, the balance of the HAT/HDAC ratio determines the acetylation of histone residues influencing gene expression (Figure 8F and Table 3). In the presence of many acetyl groups (i.e., hyperacetylation) at specific lysine residue for histone H3 and H4, the chromatin is relaxed and accessible to the transcriptional proteins, resulting in increased gene transcription; conversely, in the presence of only few acetyl gropus (i.e., hypoacetylation), the chromatin is condensed, preventing access of transcriptional proteins and resulting in gene silencing (Strahl and Allis, 2000). Similar to acetylation, the phosphorylation of histone is crucial to chromatin modifications, and activates gene transcription downstream of cell signaling events i.e., ERK, MSK1, MAPK1, IPL1. A recent study has shown that the *in vivo* acute alcohol exposure induced H3 serin-10 and serin028 phosphorylation, which was transiently increased at 1 h but decreased at 4 h after alcohol administration (Aroor et al., 2010). On the other hand, persistent H3K9 acetylation in the liver was observed at 4 h after alcohol exposure *in vivo*. Interplay between histone acetylation and phosphorylation in context with gene activation has been examined. It is clear that ethanol induces histone phosphorylation and acetylation synergistically or in independent pathways to regulate target gene expression. Moreover, changes in the epigenetic state of chromatin may have affected an appropriate transcriptional response by chronic alcohol exposure. Specifically, a recent study (Pascual et al., 2009) noted that receptor subunit changes and epigenetic mechanisms underlying the effects of adolescent alcohol exposure onsubsequent drinking behavior, this integration will require conceptualization of the problem or model at multiple level from behavioral to genetic. Further studies will be required to assess transcripteomic/proteomics and metabolomics approaches which may facilitate a better understanding of the biological role.

## Ethanol effect on transcriptional factors

**Figure 9:**
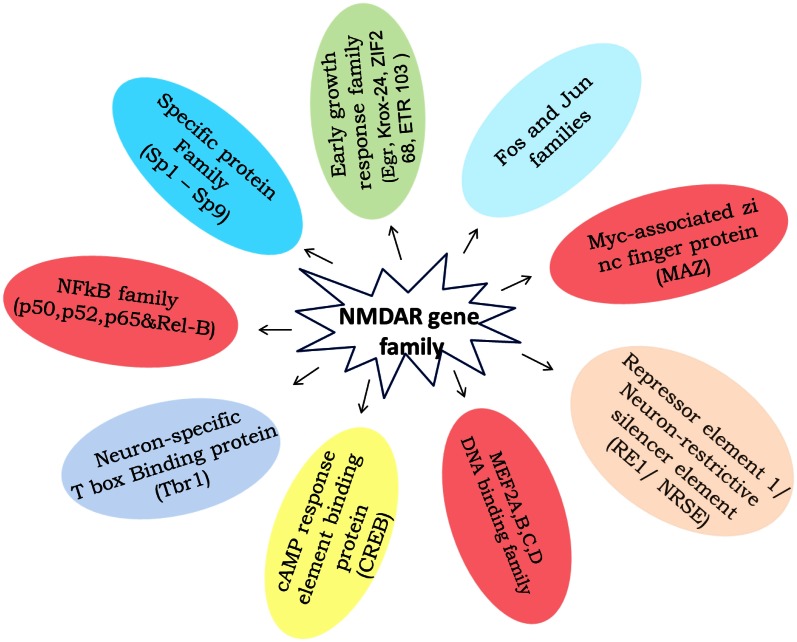
**The diagram summaries the ethanol induced to NMDA receptor subunits transcriptional regulatory binding protein family expression.** Available literature characterizing the effect of chronic ethanol on transcription factors binding elements involving gene regulation.

Transcription factors are an attractive target category for manipulation and gene regulation in the small group of transcription factors that have been identified to bind to promoter or enhancer regulatory elements in genes that are regulated by an external stimulus/stress. The NMDA Receptor, the exact mechanism by which ethanol exerts its effect, is still a matter of debate. The processes that are involved in the regulation of transcriptional potential are varied and highly complex, and include activation and inhibition of transcription factors, modification of chromatin and DNA structure, and induction of non-coding RNAs. Although neurons contain hundreds of transcription factors [early growth response factor-Egr; signal transducers and activators of transcription-STATs; glucocorticoid receptor-GR, TNF-α; transcription factor activator protein-1 (AP-1): Jun and Fos families; Specific protein family (Sp1-Sp9); Neuron-restrictive silencer element (NRSE/RE-1); Neuron-specific T box binding protein (Tbr-1); Myc-associated zinc finger protein (MAZ); MEF2A,B,C,D-DNA binding family; cAMP response element binding protein (CREB); transcription factor activator protein (TFAP) 2β ; Nucleus accumbens 1 transcription factor-NAC1, etc.], a complex combination of context-specific modifications and cell type specific transcriptional activators (Figure 9) and co-activators could enable NMDAR to coordinate the many neurobiological network processes described here and undoubtedly others which remain to be identified. Indeed, it seems that in an investigation of any given process, simple or complex physiological or pathological, one is probably justified in assuming that NR dependent gene expression is more likely to be involved.

Most researcher demonstrated various mechanisms for alcohol-induced modulation of NR genes have promoters that consist of the TATA box close to the 5′ end of the gene and, farther upstream, several motifs recognized by specific transcription factors. Early growth response factor 1 (Egr-1, also known as nerve growth factor induced A: NGFI-A, Krox-24, ZIF268, ETR 103, and TIS8) is a zing finger transcription factor discovered for its role in the regulation of cell growth and proliferation (Derdak et al., 2012). Synthesis of Egr-1, an immediate early gene is rapidly and transiently induced in response to a variety of stimuli (cytokine, growth factors) as well as environmental stress (ischemic stress, seizure, tissue damage, etc.). Transcription of Egr-1 is the primary mediator for increased Egr-1 DNA binding activity, although reports of post-transcriptional and post-translational regulation have suggested a more complex regulatory process. The mechanism for increased ERK1/2 activation and Egr-1 production are not known, but may involve ROS. From this, we understand that increased activation of the ERK1/2-Egr-1 pathway may be an important contributor to the increased sensitivity of ethanol exposure animal model. However, *in vivo* studies testing this hypothesis have not yet been reported.

AP-1 (activating protein-1) is another important transcription factor that is regulated by BDNF (Figure 7). AP-1 proteins consist of a family of Jun/Fos dimers that include different Jun proteins (c-Jun, JunB, and JunD) and Fos proteins (c-Fos, FosB, Fra-1, Fra-2, and FosB2) (Shaulian and Karin, 2002). The binding of the Fos-Jun heterodimer to the DNA element known to be the AP-1-binding site is redox-regulated. This means that enhanced free radical activity could have a direct bearing on the function of this transcription factor. AP-1 regulates the transcription of genes with a consensus DNA recognition sequence TGA/(C/G), designated as 12-*O*-tetradecanoylphorbol-13-acetate (TPA)-responsive element (TRE) in their promoter region. The mechanism for this activator is not clearly defined, but may be related to alcohol metabolism and activation of mitogen-activated protein kinase, JNK and ERK1/2 with response to ethanol exposure, and may also regulate the transcription of matrix metalloproteases and collagen type I.

The CREB family comprises CREB, CREM, CRE, and ATF1 (activation transcription factor-1) which can be homo or heterodimer to the same *cis*-regulatory element (CRE). In the nervous system, the CREB target includes, genes for the neurotrophin (BNDF) brain-derived nerutotorphic factor (Nagy, 2008), the NMDAR coupled signaling molecules nNOS (Sasaki et al., 2000), the circadian clock regulator Per1, and the Alzheimer disease related protein presenilin (Mitsuda et al., 2001). Conversely over expression of a dominant-negative CREB in different regions reduces certain types of long-term depression, and alter the long-lasting behavioral plasticity that is associated with ethanol drug addiction (Nestler, 2001). However, phosphorylated CREB significantly changes the DNA binding of the CRE site by a nuclear extract following the ethanol-induced up regulation of the NR2B gene. In short, well-documented evidence linking the CREB family most important role is to promote cell survival during embryonic development and in the adult brain.

## Ethanol effect on translational and post-transcriptional changes

The term “regulation” has been used to encompass single and multiple controlling and/or co-coordinating events of unknown configuration that involve any number of combined molecular processes, presumably leading to global gene expression outcomes. Therefore, it is important to understand ethanol inter-related aspects of gene regulation and to account for both kinds of upstream and downstream of neuronal translation. NMDA receptor activation may play a role in the translocation of translational components to areas of synaptic activity and mRNA into new protein synthesis. In general the synthesis of new polypeptides encoded by mRNAs is an essential process [(1) Initiation: 43S pre-initiation complex and 40S with eIF1A, eIF2, eIF3; (2) elongation: peptidyl tRNA with eEF1A,B, eEF2; (3) termination or release: stop codon UAG with eRF1,2,3] involved in nearly every aspect of cellular survival and growth.

Considerable data have been generated documenting the importance of the number of signaling cascades are active in response to calcium entry at the NMDA receptor. Reasonably strong evidence indicates that the NMDAR activation is the ERK (extracellular signal-regulated kinase) signal transduction pathway (Thompson et al., 2007) and regulation of mTOR signaling and protein synthesis (Kaphazan et al., 2007). A leading hypothesis suggests that the translation of mRNA containing structured 5′-UTRs might be selectively modulated through the regulation of helicases capable of relieving structural impediments (Liu et al., 2004). Indeed, evidence of this mechanism involving control of specific translation initiation factors supports this hypothesis. More direct evidence linking NMDAR activation to the repression of protein synthesis reveals that NMDAR regulation of translation elongation (eEF2 kinase via Rapamycin pathway) and termination (eRF via GTP hydrolysis process) may in fact be representative of a more finely regulated mechanism that contributes to the proper timing and spatial localization of neuronal translational and terminal events (Browne and Proud, 2004; Kapp and Lorsch, 2004).

In culture cortical neurons NMDAR activation stimulates Auroa kinase to phosphorylate CREB and subsequently enhance CaMIIα (the α subunit of calcium-calmodulin-dependent protein kinase II) mRNA polyadenylation (Huang et al., 2007). Another important hallmark is the events leading to CREB linking with FMRP (Fragile × mental retardation protein) and translational suppression or inhibit synaptic translation via its involvement in the micro RNA (miRNA) pathway (Filipowicz et al., 2005). Other major regulatory events influencing the activity of NMDARs are the post-translational modifications, especially the phosphorylation state of the receptor. NR2A, NR2B are phosphorylated on serine and tyrosine residues, and the alternatively spliced isoform of the NR1 subunits is phosphorylated on serine residues (Ferreira et al., 2011). Recently, evidence has emerged that the activity of several kinase (Fyn, Src, PKA, PKC, CaM kinase II, CDK5) and phosphatatese (PP1, PP2B) on NMDAR subunits' phosphorylation was shown to be affected by ethanol, leading to altered ethanol sensitivity and or functional activity of NMDARs (Nagy, 2003, 2008).

### Trans-acting factors as regulators of translation

**Figure 10:**
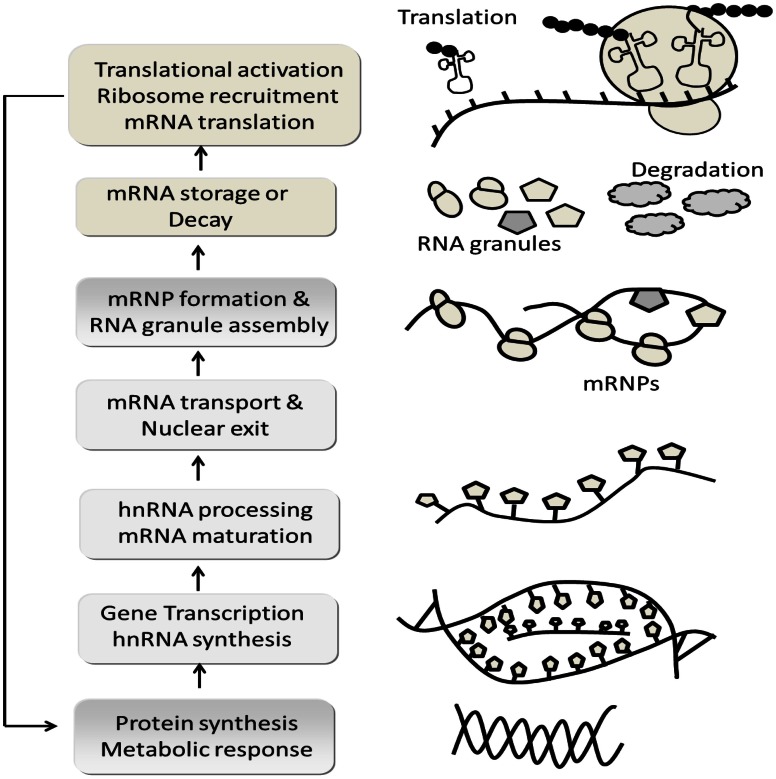
**Schematic flow chart depicts, complex mRNA life cycle.** In generally the expression of biologically active proteins in eukaryotic cells is governed by multiple events: chromatin structure, transcriptional initiation, processing and modification of mRNA transcripts (mRNA is synthesized as precursor heteronuclear RNA: hnRNA and change into mature mRNA molecules), transport of mRNA into cytoplasm (hnRNA processing occurs largely in the nucleus and is accompanied by the binding of mRNA with numerous RNA binding proteins that form messenger ribonucleoprotiens; mRNP), stability or decay of mRNA transcripts, initiation and elongation of mRNA translation, co/post-translation modification and intercellular transport and degradation of the expression protein. The model was adapted and modified from Adeli (2011).

There was significant progress in our understanding of the impact of spatially regulating translation in neurons with response to ethanol. In general, the translation machinery mechanism has a complex life cycle of mRNA and is associated with key regulatory events (Figure 10). The presence of a start-site consensus sequence, secondary structure, upstream open reading farm (ORFs) or AUGs, terminal oligopyrimidine (TOP), and internal ribosomal entry site (IRES) are all motifs within an mRNA that can determine translational efficiency. RNA regulatory domains are referred to as *cis*-acting elements (*cis*-elements consist of enhancers/promoter, silencer, insulator/boundary elements and locus control regions) within an mRNA sequence that interact with specific RNA binding proteins called trans-acting factors. For many genes, their tissue and developmental expression is regulated by the presence of positive and negative *cis*-regulatory elements in their 5′ flanking region, upstream of the transcriptional start site. Much attention has been focused recently on RNA-protein interactions and mRNA stability or mRNA decay having key role in gene expression. Several mRNA-binding proteins that have been identified (Pesole et al., 2001) as key factors involved in the regulation of mRNA stability (Guhaniyogi and Brewer, 2001; Anji and Kumari, 2006), are associated with mRNA during their transport from the nucleus to the cytoplasm, or to their site of translation (Lawerence and Singer, 1986; Shiina et al., 2005). Taken together, this co-ordinated orchestration of related mRNA through turnover, localization and/or translatability provides the cell an agile means to rapidly adapt to its every changing environment.

Interestingly, recent finding from Anji and Kumari (2011) demonstrated that a *cis-acting* region in the NMDA receptor NR1 subunit 3′-UTR interacts with the novel RNA-binding protein's beta subunit (88 kDa) of alpha glycosidase II (116 kDa) and annexin-A2 (37 kDa) with response to chronic ethanol exposure *in vivo* in the adult cerebral cortex region (Anji and Kumari, 2006, 2011). Both *in vivo* and *in vitro* studies implicate interactions between the *cis*-acting region, Δ4 RNA and trans-acting proteins (GIIβ, GIIα and Annexin A-2) as important determinant of the ethanol-regulation of the NR1 subunit's gene expression. Further molecular characterizations of these three proteins will answer many more questions. Similar studies in neurons and other polarized cells have led to the identification of 3′UTR sequences important in targeting mRNAs to specific regions within the cytoplasm, allowing localized protein synthesis (Grillo et al., 2010). In addition to 3′UTR *cis*-regulatory elements is the AU-rich element (ARE), and small RNA/RISC mediated changes in mRNA stability are complex and incompletely understood.

## Genetically altered mouse models of iGlu receptor subunits

The development of the reverse genetic approach such as transgene expression and gene knock-out, has greatly contributed to the elucidation of the mechanism of specific biological functions, in particular in studies examining the involvement of specific proteins in the molecular mechanisms of synaptic plasticity, learning, and memory. In this section we will discuss briefly about the loss of function through specific NMDA receptor subunit deletion, either by germ line or conditional knock-out, or by RNA interference (RNAi). As RNAi therapeutics become a clinical reality, the long-term implications of these unintended off-target effects will need to be better understood and controlled.

### NR1 gene knockout studies

It is well-known that chronic, excessive consumption of alcohol can cause brain damage/structural changes in the regions important for neurocognitive function. In knockout mice, the NR1 gene was disrupted by the insertion of a neo cassette in the first exon of the gene, while in the other approach, exons 12–19 were deleted and replaced with a neo cassette (Li et al., 1994). In both ways, the inactivation of the NR1 gene led to total loss of the NR1 protein and resulted in perinatal lethality. Targeted deletion of the essential NR1 subunit resulted in ablation of all NMDA receptor function in mice and can be used to study NMDA receptor mediated aspects of development. Whisker-related somatosensory patterning fails to develop in mice lacking NR1, with an insufficient level of NR1, or deficient in calcium permeability and in magnesium block, and thus coincidence detection (Iwasato et al., 1997). The cortex-restricted knockout of NR1 resulted in a similar lack of refinement of primary somatosensory cortical dendrite arbors and thalamo-cortical axon arbors and loss of patterning (Iwasato et al., 2000; Lee et al., 2005). Calarco et al. (2011) clearly demonstrate that the brain-specific regulation of alternative splicing is at least as precisely regulated as transcription. *In vivo* studies have shown that the brain-specific RNA-binding protein Nov2 is required for the inclusion of the NR1 exon 19, as this exon is already included in the NR1 mRNA detected Nova2^−/+^ fore brain. Evidence from mini-gene assay in cell cultures suggests that uibquitious heterogeneous nuclear ribounucleoproteins A1 and H (hnRNP A1 and hnRNPH) and neuroblastoma apoptosis-related RNA-binding protein (NAPOR, also called CUGBP2 or ETF-3) also regulate NR1 exon 19 inclusion (Li et al., 2001; Zhang et al., 2002). Loss of forebrain-specific splicing of NR1 exon 19 in Nova2^−/+^ brain thus suggest that the combinatorial control of the NR1 exon 19 acts in a cooperative (Ule and Darnell, 2001) rather than additive manner, allowing the activity of a single factor to cause a major splicing changes.

### NR2 gene knockout studies

Formal proof of the causative role of NMDA receptor subunits and crucial additional insight into the associated disease mechanisms/phenotypes were provided by the analysis of naturally occurring and genetically engineered neurological animal mutants (Granger et al., 2011). To study the functional roles of NMDA receptor subunits, NR2A–NR2D genes have been disrupted by gene targeting. Targeted disruption of NR2B results in the early death of the mutant mice (NR2B^−/−^), demonstrating that NR2B is essential for the survival of newborn mice. Although knockout animal have no gross anatomical abnormalities in the brain, suckling responses are impaired and appear to be the cause of death (Kutsuwada et al., 1996). Tactile hairs (whiskers) on the snout of rodents are arranged in an array and collectively form a unique sensory organ. This sensory organ is connected to the brain via the trigeminal nerve, and in the brainstem, trigeminal nuclei whisker-specific neural patterns, termed barrelette, are formed. The barrelettes are absent in the NR2B^−/−^ mice, indicating that NR2B are involved in the development of neural networks in the CNS (Kutsuwada et al., 1996). In contrast, overexpression of the NR2B subunit in the brain of transgenic mice resulted in an enhancement of the activation of NMDA receptors and improved learning and memory on various behavioral tasks, suggesting an essential role for NR2B in cell-cell transmission underlying memory functions (Tang et al., 1999). NR2D knockout mice (NR2D^−/−^) grow normally and no obvious histological abnormalities were found in the various brain regions or in the formation of barrelettes. Interestingly, NR2D ablation results in alteration of monoaminergic neuronal systems in the adult hippocampus, due to increased DA and 5-HT metabolism. These changes are not a consequence of an altered expression of the other NMDA receptor subunit in the hippocampus, nor to obvious histological abnormalities in any brain region (Ikeda et al., 1995; Miyamoto et al., 2002). In the elevated plus-maze test and in the light-dark box test, NR2D^−/−^ mice revealed a more exploratory behavior than wild-type mice (Miyamoto et al., 2002) suggesting that NR2D^−/−^ mice may have a reduced susceptibility to stress and/or reduced psychological anxiety. In particular, the silencing of NR2A and NR2C-subunits resulted in significant reduction of the NMDA receptor channel current and LTP at the hippocampal CA1 synapses (Ebralidze et al., 1996). But deficient NR2A^−/−^ subunits alone showed only a moderate impairment in spatial and contextual learning and a slight reduction in the NMDA receptor channel current.

To generate mice lacking both NR2A and NR2C, NR2A and NR2C, mutant mice were mated with each other (Kadotani et al., 1996). The NMDA receptor-mediated components in cerebellar granule cells are virtually abolished in mice lacking both NR2A and NR2C. NR2A or NR2C ablation in mice does not affect motor coordination and animals retain the ability to manage simple coordinated tasks, such as walking on the ground, staying stationary, or slowly running on the rota-rod. However, NR2A^−/−^/NR2C^−/−^ mice reveal difficulties in more challenging tasks such as walking on the narrow bar or staying on the quickly running rota-rod (Kadotani et al., 1996).

### NR3 subunits knockout studies

The NR3A and NR3B knockout mice are fertile and survive until adulthood without any noticeable abnormality, consistent with the proposed modulatory role for the NR3 subunits (Niemann et al., 2007). Cell cultures using NR3A genetically modified mice also provided some clues about NR3A physiology. Whole-cell NMDA receptor currents in NR3A^−/−^ neuronal cortical cultures are unchanged, but current density is increased (Wee et al., 2000). In NR3A^−/−^ retinal cells NMDA receptor mediated [Ca^2+^] rises were significantly higher than those in control cells. Conversely, a population of neurons overexpressing NR3A has less conductance and show less Ca^2+^ permeability to NMDA-induced currents.

NR3A confers neuroprotection against several forms of excitotoxic insults. Cultured neurons prepared from NR3A^−/−^ mice display greater sensitivity to damage by NMDA than control neurons (Nakanishi et al., 2009). *In vivo*, adult wild-type mice and neonatal NR3A KO mice suffer more damage than neonatal mice, which contain NR3A, after hypoxia–ischaemia (Nakanishi et al., 2009). In addition, in P19 NR3A^−/−^ mice, the cortical spine density was about threefold higher than in normal mice, which suggests a link between NMDA receptor mediated current aptitudes and spine density (Myers et al., 1999). It is suggested that, together this data supports a neuroprotective role for NR3A. The silencing of the NR3B subunit causes mild deficiencies in motor learning and coordination in mice. These mice also have higher social interactions in the cage, showing anxiety-like behavior, suggesting a possible link of NR3B with psychiatric disorders (ALS) in humans (Chatterton et al., 2002; Nishi et al., 2001; Niemann et al., 2007), because NR3B subunits are predominately expressed in motor neurons. However, contradictory to the putative neuroprotective role of NR3B, no motor neuron death was observed in knockout mice (Niemann et al., 2007). Consistently, approximately 10% of the human European- American population has a complete deficiency in NR3B, which is not associated with ALS or with any obvious clinical problems (Niemann et al., 2008). This is suggests that wider expression, a pathogenic role for NR3B in the context of motorneuron diseases can be excluded.

In particular, the development of reverse genetic methods such as transgene expression and gene targeting has allowed substantial progress in the understanding of the molecular mechanisms of cell–cell and cell-extracellular interactions. The various examples of the NMDA receptor subunit gene knock-out described above, illustrate the variety of phenotypes that may be produced by a deficiency of a specific type of glutamate receptor subunit. Depending on the endogenous level, the pattern of expression and the respective importance for developmental processes of each receptor's subunits, the loss of the subunit leads to early embryonic death, and mild behavioral impairment and total absence leads to neuronal multi-disorder.

## Summary

Alcohol abuse is one of the major sources of public health (social and medial) problem throughout the world. In this review, we have attempted to draw together the evidence gathered from past and present research on the effect of alcohol on the N-methyl-D-asparatic acid receptor and pathophysiological states and molecular mechanism (excitatory signaling, plasticity, gene regulation, protein synthesis etc.). Acquiring detailed understanding of the effects of alcohol in the central nervous system is a critical factor in resolving this problem. Alcohol can alter the function and structure of both the developing and adult brain, causing multiple neurological disorders (cognitive dysfunction, ischemia, epilepticus, learning and memory and seizures). Numerous preclinical diagnostic imaging studies (CT, MRI, DTI, CSF, PET, SPECT techniques) have revealed that cortical and subcortical, frontal lobe, and hippocampal regions significantly shrink and there is a loss of cholinergic cells in the basal forebrain, frontal cortical gray and white matter. In addition, there is a decrease in the amounts of N-methyl-D-aspartate acid receptors (NMDAR) in the frontal lobe, and a measure of neuron levels also illustrated frontal lobe degeneration in alcoholics. The molecular action of alcohol on the brain is complex, and involves numerous mechanisms and signaling pathways. Increased oxidative stress (NOS, ROS) causes energy deficiency which leads to the dysfunctioning of presynaptic neurotransmitter release. As a result, there is net increase in extracellular glutamate, causing neurotoxicity and apoptosis. There are few studies demonstrating significantly decreased levels of Gap-43 (hall mark of growth associated neuronal protein) and protein in the cerebellum and increased levels of mRNA and protein in the hippocampal region on exposure to ethanol.

It is important to note that, previous studies showed that ethanol attenuates the NMDA receptors function in dose-related fashion *in vitro* and *in vivo*. Mounting evidence reveals that immunocytochemical and biochemical studies have shed light on the distribution of the NMDA receptor molecules on the neuronal cell surface and the relationship between subunit assembly and cell surface expression. Currently, a growing body of evidence supports an interaction between ethanol and the NMDA receptor's (NR1, NR2, and NR3 genes) expression in the spatiotemporal region of brain and its effects in several intercellular signaling pathways (neurotropic factors). This includes links between several events which are co-ordinated with transcription (transcriptional regulatory factors), RNA translational, post translation modification (mRNA splicing, RNA editing, and mRNA stability) and translocation. The physical linkage of these receptors and signaling molecules (kinase, phosphatases) is an important way to facilitate rapid modulation of the receptor in response to post-synaptic activity. Recently, Anji and Kumari (2011) acquired data to support this hypothesis—ethanol-mediated post-transcriptional regulation of NR1 mRNA in *in vivo* (adult brain) and *in vitro* (fetal cortical neuron) studies. The interaction of NR1 mRNA with GII β and other trans-acting protein may result in NR1 mRNA stabilization, a process that in turn may increase the NR1 polypeptide levels, an indirect effect on NR1 mRNA translation. The majority of NR1 gene splices' variants exhibit heterogeneity with respect to agonist and antagonist affinity, zinc modulation and regional development expression patterns. However, significant progress has been made in understanding the mechanism of constitutive NMDA receptor splicing. On the other hand, a disadvantage of this mechanism is the mis-regulation of pre-mRNA processing that is apparent in pathophysiological diseases. Recent studies showed Kainate receptor also contribute to the to the anxiolytic effects on ethanol (Fetal alcohol spectrum disorders), and ethanol inhibition of AMPA receptors along with other glutamate receptors. But still, several intriguing questions remain unanswered. Alternatively, another tool for the loss-of-function approach in transgenic/gene-targeting technology has become the standard methodology for investigating the functions of NMDAR subunits genes in a complex biological system.

Despite the huge progress, there is a lot left to understanding the molecular mechanism underlying the effect of alcohol on highly regulated cellular pathways. Future advances will come from the application of technologies that allow an integrative analysis of multiple signaling pathways and transcriptional factors. Proteomics techniques, such as Mass spectrometry, should aid in the characterization of signaling and transcriptional complexes, and in the identification of activity-induced post-translational medications. In addition, metabolomics (metabolite profiling, metabolic fingerprinting) and RNA-seq approaches may contribute to alcohol-associated research, which may define the role of many different metabolic pathways affected by alcohol. The ultimate goal of this research is to understand transcription, post-transcriptional, translational and post-translational mechanisms, and underlying alterations in NMDAR, induced by alcohol.

### Conflict of interest statement

The author declares that the research was conducted in the absence of any commercial or financial relationships that could be construed as a potential conflict of interest.
